# Known and novel viruses in Belgian honey bees: yearly differences, spatial clustering, and associations with overwintering loss

**DOI:** 10.1128/spectrum.03581-23

**Published:** 2024-06-11

**Authors:** Ward Deboutte, Lina De Smet, Marleen Brunain, Nikolas Basler, Riet De Rycke, Lena Smets, Dirk C. de Graaf, Jelle Matthijnssens

**Affiliations:** 1 KU Leuven–University of Leuven, Department of Microbiology, Immunology and Transplantation, Division of Clinical and Epidemiological Virology, Rega Institute, Leuven, Belgium; 2 UGent–Ghent University, Department of Biochemistry and Microbiology, Laboratory of Molecular Entomology and Bee Pathology (L-MEB), Ghent, Belgium; 3 VIB Center for Inflammation Research and BioImaging Core, Ghent, Belgium; 4 Department of Biomedical Molecular Biology, UGent–Ghent University, Ghent, Belgium; USDA-ARS National Center for Cool and Cold Water Aquaculture, Kearneysville, West Virginia, USA

**Keywords:** bee viruses, pollinator decline, bee health, colony losses, winter survival

## Abstract

In recent years, managed honey bee colonies have been suffering from an increasing number of biotic and abiotic stressors, resulting in numerous losses of colonies worldwide. A pan-European study, EPILOBEE, estimated the colony loss in Belgium to be 32.4% in 2012 and 14.8% in 2013. In the current study, absolute viral loads of four known honey bee viruses (DWV-A, DWV-B, AmFV, and BMLV) and three novel putative honey bee viruses (Apis orthomyxovirus 1, apthili virus, and apparli virus) were determined in 300 Flemish honey bee samples, and associations with winter survival were determined. This revealed that, in addition to the known influence of DWV-A and DWV-B on colony health, one of the newly described viruses (apthili virus) shows a strong yearly difference and is also associated with winter survival. Furthermore, all scrutinized viruses revealed significant spatial clustering patterns, implying that despite the limited surface area of Flanders, local virus transmission is paramount. The vast majority of samples were positive for at least one of the seven investigated viruses, and up to 20% of samples were positive for at least one of the three novel viruses. One of those three, Apis orthomyxovirus 1, was shown to be a genuine honey bee-infecting virus, able to infect all developmental stages of the honey bee, as well as the *Varroa destructor* mite. These results shed light on the most prevalent viruses in Belgium and their roles in the winter survival of honey bee colonies.

**IMPORTANCE:**

The western honey bee (*Apis mellifera*) is a highly effective pollinator of flowering plants, including many crops, which gives honey bees an outstanding importance both ecologically and economically. Alarmingly high annual loss rates of managed honey bee colonies are a growing concern for beekeepers and scientists and have prompted a significant research effort toward bee health. Several detrimental factors have been identified, such as varroa mite infestation and disease from various bacterial and viral agents, but annual differences are often not elucidated. In this study, we utilize the viral metagenomic survey of the EPILOBEE project, a European research program for bee health, to elaborate on the most abundant bee viruses of Flanders. We complement the existing metagenomic data with absolute viral loads and their spatial and temporal distributions. Furthermore, we identify Apis orthomyxovirus 1 as a potentially emerging pathogen, as we find evidence for its active replication honey bees.

## INTRODUCTION

Bees, both managed Western honey bees (*Apis mellifera*) and wild bees, contribute substantially to ecosystem services through crop pollination (1). Despite the fact that the contribution of other insects to pollination efforts are understudied (2), honey bees remain the most valuable and important pollinators for crops worldwide (1, 3). Recent studies have shown that honey bee populations face the same threats that also lead to a decline of other insect species over the last years (4, 5). Numerous investigations have revealed that, despite a global net increase in managed beehives (6), regional losses of colonies are rampant (7–9). Substantial effort has been directed at elucidating the cause of these trends. Several studies have attempted to identify single causal factors underlying the declines. For example, climate change as well as habitat loss or a decrease in habitat quality (e.g., by pesticide use) have been shown to have a negative impact on pollinator health (10–12). Recently, it has become clear that these identified causal factors alone cannot explain all the reported cases of colony loss, and the underlying drivers for honey bee decline are multifactorial (13). These drivers include the aforementioned environmental variables but also include biotic variables such as (bacterial and viral) infections, parasites, and pests, the honey bees’ immune system, and beekeeper practices. None of these variables influencing bee health are independent factors; instead, many of them are strongly confounded. For example, the *Varroa destructor* parasite, a mite originating in southeast Asia that was introduced from its original host (*Apis cerana*) into Western honey bees and subsequently spread worldwide (14), is a vector of several bee viruses (15). Parallel to its role as viral vector, *V. destructor* can influence the immune status of its host (16). Globalization of *V. destructor* and concomitant deformed wing virus A (DWV-A; member of the genus *Iflavirus*, family *Iflaviridae*, order *Picornavirales*) infections raised the question about which role DWV-A plays in colony health. Recent studies have revealed an association between DWV-A infections and colony health status (17–19). Deformed wing virus B (DWV-B, originally called Varroa destructor virus 1), a virus very similar to DWV-A, was initially described in the mite and has also been associated with a weak colony health status (20). While DWV-A and DWV-B are the most studied viruses in bees, other viruses have been characterized with a potentially detrimental impact on colony health, especially in combination with a Varroa mite infestation. Most of these viruses are also members of the *Picornavirales* order (which is common among insect viruses [21]), acute bee paralysis virus (22), sacbrood virus (23)*,* black queen cell virus, and Kashmir bee virus (24), among many others (25). Several studies have revealed associations with one or more viruses and the colony health status, although these associations do not seem to be universal (26). The substantial cost reduction of DNA sequencing in the last decade enabled a number of metaviromic studies which have drastically increased the number of known honey bee viruses (27–29). Still, the relevance and influence on colony health of many of these newly described viruses remain enigmatic.

In a previous study, we used a viral metagenomics approach on 100 pools of honey bee samples collected throughout Flanders/Belgium in 2012 and 2013 in the framework of the EPILOBEE project (EU funding number: NP/EFSA/SCER/2014/02) (30). This project resulted in the identification of known as well as numerous unknown and novel honey bee viruses. In the current study, the absolute viral load of four known honey bee viruses and three novel putative honey bee viruses was determined in 300 bee samples from different hives collected in the EPILOBEE framework (31). Associations between individual virus loads and winter survival status were assessed, prevalences were calculated, and the association between viruses and winter survival was evaluated using multiple logistic regression. Furthermore, the underlying geographical pattern of the viruses was evaluated. Finally, one of the newly described viruses (Apis orthomyxovirus 1) was proven to be a true honey bee virus by utilizing Multiplex Ligation-dependent Probe Amplification (MLPA) and transmission electron microscopy. RT-PCR showed the presence of this virus in all developmental stages of honey bees and in a Varroa mite parasitizing an infected pupa, as well as in all tagmata and in the hemolymph of adult workers.

## MATERIALS AND METHODS

### Selection of viruses and phylogenetic analysis

In a previously conducted metaviromic survey (30) on samples from the Flemish part of the EPILOBEE study (31), virus-like particle enrichment and Illumina sequencing were performed on 102 pools of six bees (two individuals from three colonies) sampled in autumn for 2 consecutive years (2012 and 2013). Metadata such as genotype, treatments, survival after the winter of the sampling year, and location were also collected within that survey. From the virus relative abundance matrix generated within that framework, seven viruses [DWV-A, DWV-B, Apis mellifera filamentous virus (AmFV), bee macula-like virus (BMLV), Apis orthomyxovirus 1, apthili virus, and apparli virus] were selected for absolute quantification in the current study. Included viruses were selected because of their high prevalence (DWV-A, DWV-B, apthili virus), the remarkably high relative abundance (apparli virus), or their potential biological relevance (Apis orthomyxovirus 1, BMLV, and AmFV) (Fig. S1). For phylogenetic analysis, one gene was selected for each of these viruses: the putative ribonucleotide reductase gene for AmFV, the putative polyprotein gene for BMLV, DWV-A, and DWV-B, the putative RNA-dependent RNA polymerase gene for apparli virus and apthili virus, and the *PB1* gene for Apis orthomyxovirus 1. In brief, phylogenetic trees were constructed by first aligning the respective protein sequences using MAFFT (32), L-INS-I setting, followed by automated trimming (TRIMAL [33], gappyout setting), tree reconstruction (maximum-likelihood) using automatic model selection (34), and 1,000 ultrafast bootstraps (35) implemented in IQtree (36). The resulting trees were plotted using the ggtree package (37), implemented in R version 4.1.2 (38). Protein sequences included in the phylogenetic reconstruction were based on a manually curated list of protein BLAST hits against NCBI’s non-redundant protein database.

### Samples, nucleic acid extraction, and qRT-PCR

In 2012 and 2013, two bees were sampled from each of 300 different Flemish colonies (150 per year) and pooled for further processing. Each of these pools was homogenized for 1 min in PBS, using ceramic beads (Precellys, Bertin Technologies, France) at 4,000 Hz in a tissue homogenizer (MINILYS, Bertin Technologies, France). DNA and RNA were simultaneously extracted using the RNA viral extraction kit (QIAgen, Netherlands) according to the manufacturer’s protocol. In order to acquire absolute abundances of the seven selected viruses, primers, probes, and standards for quantitative reverse transcription-PCR (qRT-PCR) were designed (Table S1). Appropriate regions for primers and probes were identified by calling single nucleotide polymorphisms (SNPs) using the sequencing data obtained in the metaviromic study (30). In brief, fastq files were aligned back to the consensus genomes of the selected viruses using bowtie2 (39), and SNPs were called using LoFreq (40), with default settings. Where possible, amplicons were chosen such that they fell in regions relatively depleted of SNPs (Fig. S7). Specificity of the primers was ensured *in silico* by blasting the primer sequences, and by Sanger sequencing the amplicons. qRT-PCR assays were run using the Taqman Fast Virus 1-step kit (ThermoFisher Scientific, USA), following the manufacturer’s instructions, on an Applied Biosystems 7500 Fast Real-Time PCR system (ThermoFisher Scientific, USA). In brief, after an activation stage of 20 seconds at 95°C, 45 replicates of 3 seconds at 95°C, and 30 seconds at the annealing temperature specific to the virus (Table S1) were run, using 5 µL DNA/RNA extract per sample. Two technical replicates were run per sample; the mean value of the two replicates were used for downstream analysis. Standard curves were created (Fig. S2), and a sample was considered to be positive if a Ct value could be determined. No Ct values could be determined in the negative controls. Ct values and absolute quantification was performed using the Applied Biosystems SDS software version 1.3.1 (ThermoFisher Scientific, USA).

### Apis orthomyxovirus 1 detection

Adult worker bees of all colonies on the experimental apiary of the UGENT campus De Sterre were sampled and tested for the presence of Apis orthomyxovirus 1 using RT-PCR. To this end, RNA was extracted using an RNA viral extraction kit (QIAgen, Netherlands) according to the manufacturer’s protocol. From the RNA extracts, cDNA was synthesized using random hexamer primers with the RevertAid H Minus First Strand cDNA Synthesis Kit (Thermo Scientific Scientific, USA), and subsequently amplified using HotStarTaq polymerase (QIAgen, Netherlands). The amplified PCR products were electrophoresed for 60 min at 100 volts through 1.5% agarose TBE gel in standard TBE buffer, stained with ethidium bromide, and visualized using UV illumination. One colony tested positive and new samples from different developmental stages were taken from that colony and subjected to RT-PCR as described above. The new samples comprised 10 worker eggs, two 3-day-old larvae, a stretched larva, two white-eyed pupae, a red-eyed pupa, a Varroa mite, its corresponding parasitized pupa, an emerging bee, and adult worker bees. The eggs were pooled together while the other samples were analyzed individually. Additionally, eight adult worker bees were separated into the different tagmata (head, thorax, and abdomen) and hemolymph was sampled to determine the infected tissues.

### Positive strand detection of Apis orthomyxovirus 1

MLPA is an amplification technique that allows strand-specific detection of RNA viruses. A specific RT-primer and probes (Table S1) were designed to detect the replicating strand of Apis orthomyxovirus 1 using the AlleleID software (Premier Biosoft, USA). RNA from Apis orthomyxovirus 1-positive adult bees were used as a template in the MLPA reactions. The RNA was extracted from complete, homogenized honey bees using the RNA lipid tissue mini kit (Qiagen, Netherlands) following the manufacturer’s recommendation. The MLPA reactions were performed as described previously using the SALSA MLPA reagents supplied by MRC Holland (Netherlands) (41).

### Transmission electron microscopy of Apis orthomyxovirus 1

All tagmata and the hemolymph of the Apis orthomyxovirus 1-positive colony were infected with Apis orthomyxovirus 1, based on the RT-PCR results. We therefore assumed that the virus was circulating in the hemolymph. Hemolymph from 50 Apis orthomyxovirus 1 RT-PCR-positive and 50 RT-PCR-negative bees was collected, and immediately, an equal volume of sodium citrate buffer (30 mM trisodium citrate, 26 mM citric acid, 0.45 M NaCl, 0.1 M glucose, 10 mM EDTA, pH 4.6) was added to prevent hemocyte coagulation (42). Hemocytes were fixed in 4% paraformaldehyde and 2.5% glutaraldehyde in 0.1M Na cacodylate buffer, pH 7.2. Low melting point agarose was used to keep the cells concentrated for further processing. The cells were fixed for 4 hours at room temperature followed by overnight fixation at 4°C after exchanging the fixative. After washing in the buffer, the cells were post-fixed in 1% OsO_4_ with 1.5% K_3_Fe(CN)_6_ in 0.1M Na cacodylate buffer at room temperature for 1 h. After washing, cells were subsequently dehydrated through a graded ethanol series, including a bulk staining with 1% uranyl acetate at the 50% ethanol step followed by embedding in Spurr’s resin. Semi-thin sections were first cut at 0.5 µm and stained with toluidine blue. Ultrathin sections of a gold interference color were cut using an ultra-microtome (Leica EMUC6), followed by a post-staining in a Leica EM AC20 for 40 min in uranyl acetate at 20°C and for 10 min in lead stain also at 20°C. Sections were collected on Formvar-coated copper slot grids. Grids were viewed with a JEM 1400plus transmission electron microscope (JEOL, Tokyo, Japan) operating at 60 kV.

### Statistical analysis

All statistical analyses were performed in python (version 3.11). Differences between viral loads were calculated using the Mann-Whitney U-test (two-tailed) implemented in scipy (version 1.10) (43), corrected for multiple testing using the Benjamini-Hochberg procedure. Multiple logistic regression was performed in R (version 4.2.2) (38) with the “Train” function implemented in Caret (version 6.0) (44) using 10-fold cross validation. Three different regressions were performed, either the “full model with first order interactions” [with formula: ~year + (dwva + aov + apthili + bmlv + apparli + amfv + dwvb) (2)], an “associated model with first order interactions” [with formula: ~year + (dwva + apthili + dwvb) (2)], and a “simple model” (with formula: ~year + dwva + aov + apthili + bmlv + apparli + amfv + dwvb). Performance of these regressions were evaluated using the confusionMatrix function implemented in Caret, and the prediction and performance functions implemented in ROCR (version 1.0) (45). Moran’s I statistic was calculated using the Moran function implemented in the pysal (version 2.1.0) and esda library (version 2.4.3) (46) using Rook contiguity weights on commune level. The geographical data for Flanders were downloaded from GADM (47). Spearman correlations were calculated using the “spearmanr” function implemented in scipy (version 1.10) (43). The metaviromic study was performed on pools of three samples (containing two individuals each). Of the three corresponding samples assayed via qPCR, the one with the highest value was used for comparison with the normalized counts. Visualizations were made using the Seaborn (version 0.11.2) (48), Geopandas (version 0.12.2) (49), Geoplot (version 0.5.1) (50), and Matplotlib (version 3.6.2) (51) libraries.

## RESULTS

### Phylogenetic analysis of the seven most prevalent honey bee viruses reveals three new taxa

**Fig 1:**
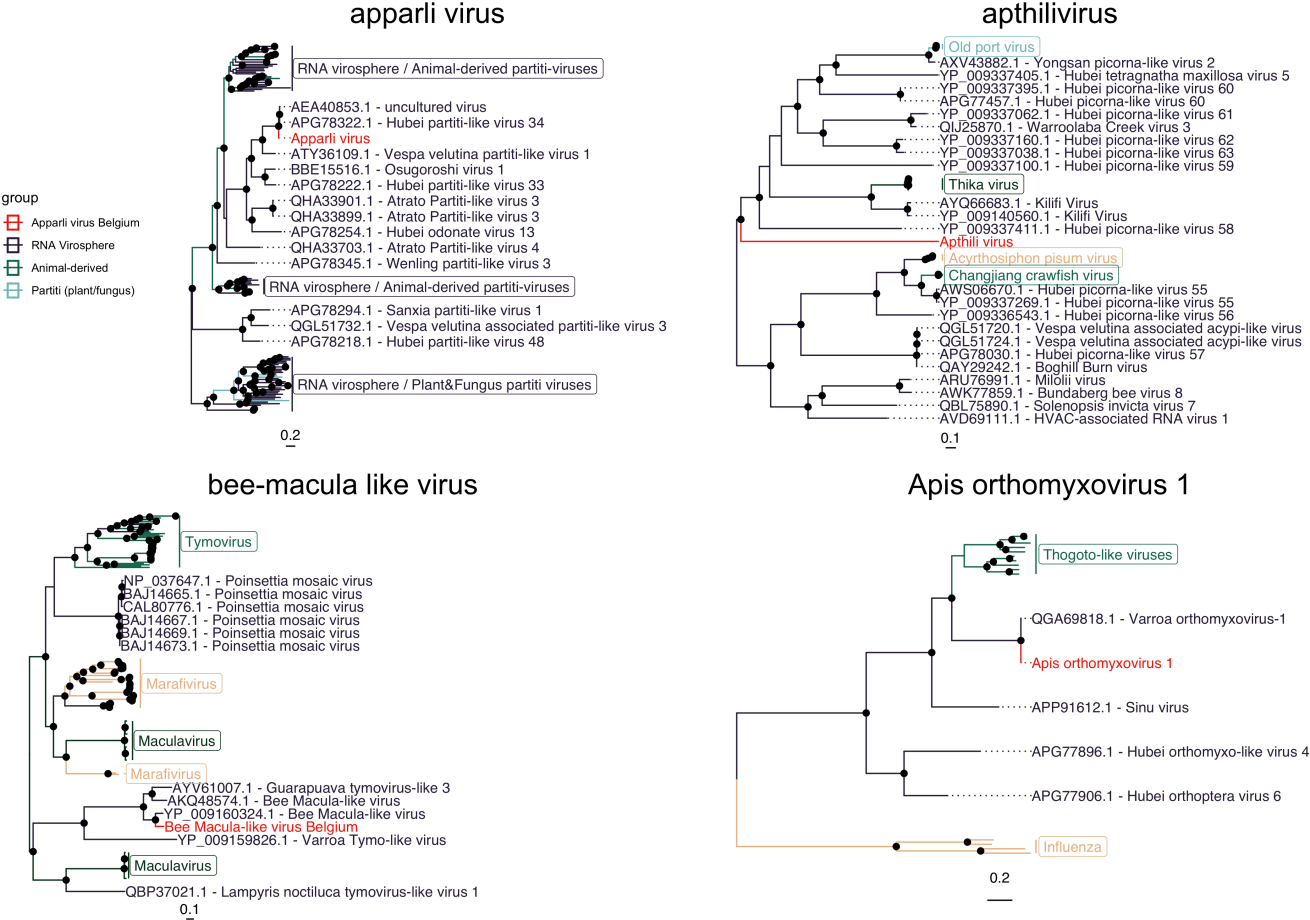
Maximum-likelihood phylogenetic trees for the newly described viruses included in this study. The viruses from this study are highlighted in red. Other relevant families, genera, species, or viral groups are indicated using other colors and a legend in case of non-clearly defined taxa, or other colors and a clade label (with rectangles) in case of clearly defined taxonomic groups. Black node labels reflect bootstrap values greater than 70. The apparli virus and apthili virus trees are based on the putative RNA-dependent RNA polymerase gene, the BMLV tree is based on the putative polyprotein gene, and the Apis orthomyxovirus 1 tree is based on the *PB1* gene. All trees are based on amino acid alignments.

The seven viruses that were selected based on their relative abundance, prevalence and potential biological relevance (Fig. S1; see Materials and Methods) contain four well-characterized viruses (DWV-A, DWV-B, AmFV, and BMLV), and three recently discovered divergent viruses. We tentatively name these three apparli virus (Apis partiti-like virus), Apis orthomyxovirus 1, and apthili virus (Apis thika-like virus) (Fig. 1; Fig. S4 through S6). As the name suggests, apparli virus was predicted (based on amino acid sequence homology) to be a partiti-like virus. Despite these viruses being known as plant- or fungus-infecting viruses (52), the obtained genomic sequence clustered most closely to partiti-like viruses derived from honey bees (AEA40853.1) and from land snails (APG78322.1) (53) (Fig. S4). Apthili virus*,* a predicted picorna-like virus, is very distantly related to a clade containing thika viruses (a picornavirus described in *Drosophila*) and Old Port viruses (a picornavirus described in arthropods) (Fig. S5). Despite the fact that the RNA-dependent RNA polymerase (RdRP) was very distantly related (25.6% amino acid identity), the capsid and the other predicted hypothetical protein were more similar to a partial viral sequence retrieved from bumble bees (MH614316.1) (Fig. S5). Apis orthomyxovirus 1, a member of the *Orthomyxoviridae*, was nearly identical (with the exception of the putative glycoprotein segment) to a virus discovered in a metaviromic survey on the *V. destructor* parasite (54), and is distantly related to the thogoto-like viruses (members of the *Orthomyxoviridae*-infecting ticks and mammals) (Fig. S6). Overall, the amino acid similarities for the three recently discovered viruses ranged from 25.63% (putative RdRP, apthili virus) to 99.69% (putative PB2, Apis orthomyxovirus 1) (Fig. S7).

### Viral load determination reveals several associations with colony winter survival

**Fig 2:**
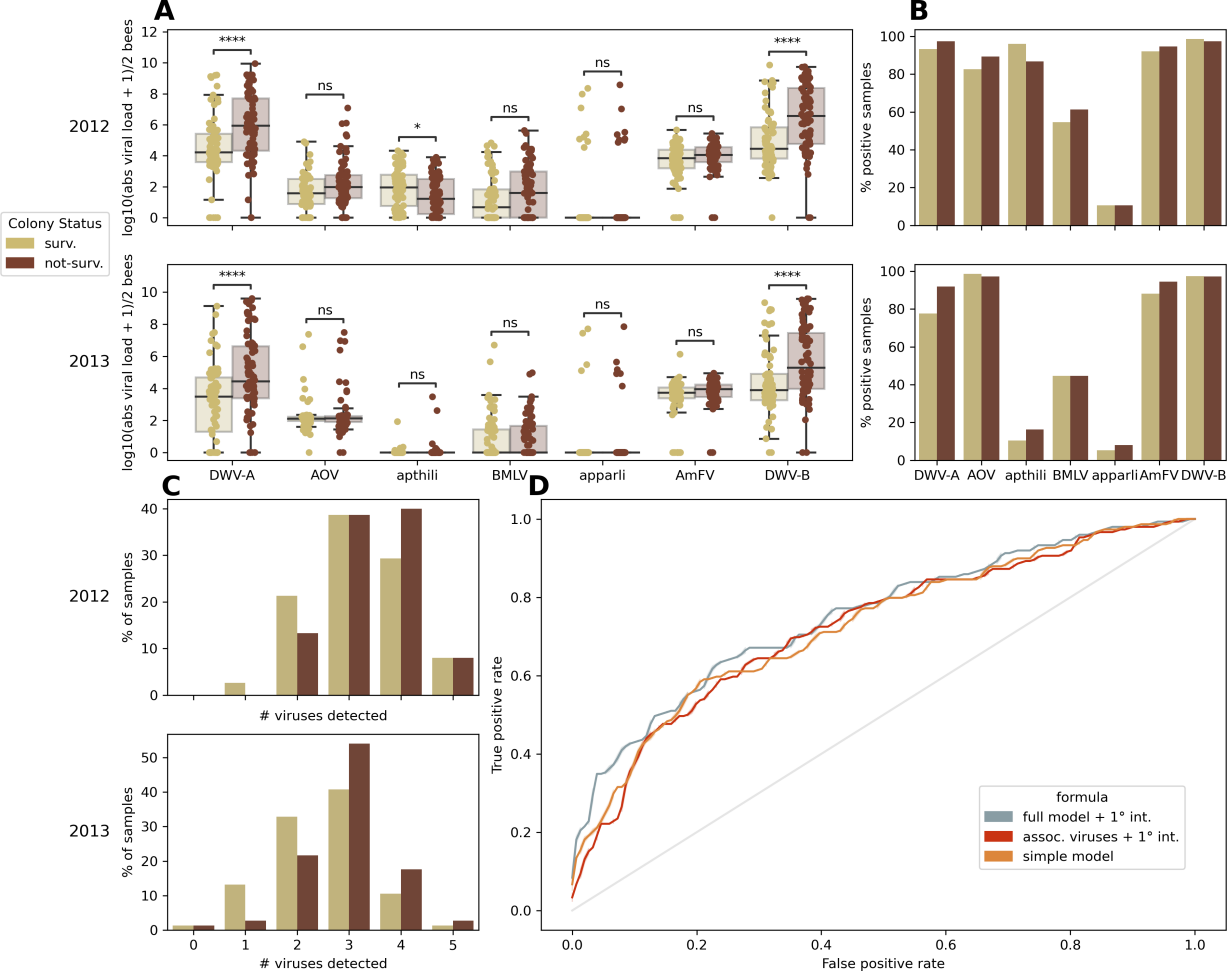
Absolute viral load quantification reveals associations with colony winter survival and yearly differences of the included viruses. Samples (with two bees per colony) from surviving colonies are indicated in light brown, those from not-surviving colonies are indicated in dark brown. (**A**) Log-transformed absolute viral loads are plotted in function of the different included viruses, either for sampling year 2012 (top, 150 samples) or sampling year 2013 (bottom, 150 samples). Significant differences (as assessed by Mann-Whitney U-tests, corrected for multiple testing with Benjamini-Hochberg correction) are indicated with asterisks above the plots. Dots reflect the different samples, and all samples are drawn. Boxes indicate the three quartile values, and whiskers extend to 1.5 interquartile ranges of the lower and upper quartile. AOV is the abbreviation for Apis orthomyxovirus 1. (**B**) Percentage of positive samples per virus are indicated. Positive samples are samples where a Ct value could be determined. (**C**) Percentage of samples versus the total number of viruses detected per sample. (**D**) Receiver operating characteristic (ROC) curves for logistic regression results using either a full model with interaction terms (blue), a reduced model containing DWV-A, apthili virus, and DWV-B (significant in panel **A**) with interaction terms (red), or a full model without interaction terms (orange).

The absolute viral loads of the aforementioned selected viruses were quantified via qRT-PCR in honey bees from 300 Flemish colonies sampled in 2012 and 2013. The viral loads of DWV-A and DWV-B were significantly higher in samples derived from colonies which did not survive the following winter, both in 2012 and 2013 (Fig. 2A). Furthermore, a significant difference in apthili viral loads between surviving and non-surviving colonies was observed in 2012. In contrast to samples from 2012, nearly all colonies sampled in 2013 were apthili virus negative (Fig. 2B). Additionally, apthili virus is the only scrutinized virus where viral loads were significantly higher in colonies surviving winter. Regardless of the high prevalence of Apis orthomyxovirus 1, BMLV, and AmFV (Fig. 2B), no significant difference in viral loads could be detected between colonies surviving and not-surviving the winter (Fig. 2A). One to three of the selected viruses could be detected by the qPCR assays in the vast majority of samples (Fig. 2C), although these numbers are heavily driven by DWV-A, DWV-B, and AmFV (Fig. 2B). To further elucidate the relation between colony winter survival status and viruses present, multiple logistic regression was performed using either a full model (including year, the different viruses, and their first order interactions), a reduced model (including year, DWV-A, DWV-B, and apthili virus and their first order interactions), or a full model without first order interactions (simple model). These regression analyses were validated using 10-fold cross validation. All three analyses performed very similarly (Fig. 2D) with accuracy values 66.00% (±5.51%), 66.67% (±5.48%), and 69.67% (±5.35%) for the simple model, reduced model, and full model, respectively. Interestingly, the sensitivity was substantially higher (in all three models) than the specificity. The significance of the included coefficients differs quite drastically between the different models, despite them performing very similarly in terms of accuracy (Fig. S8).

### Spatial analysis reveals that viral loads cluster geographically

**Fig 3:**
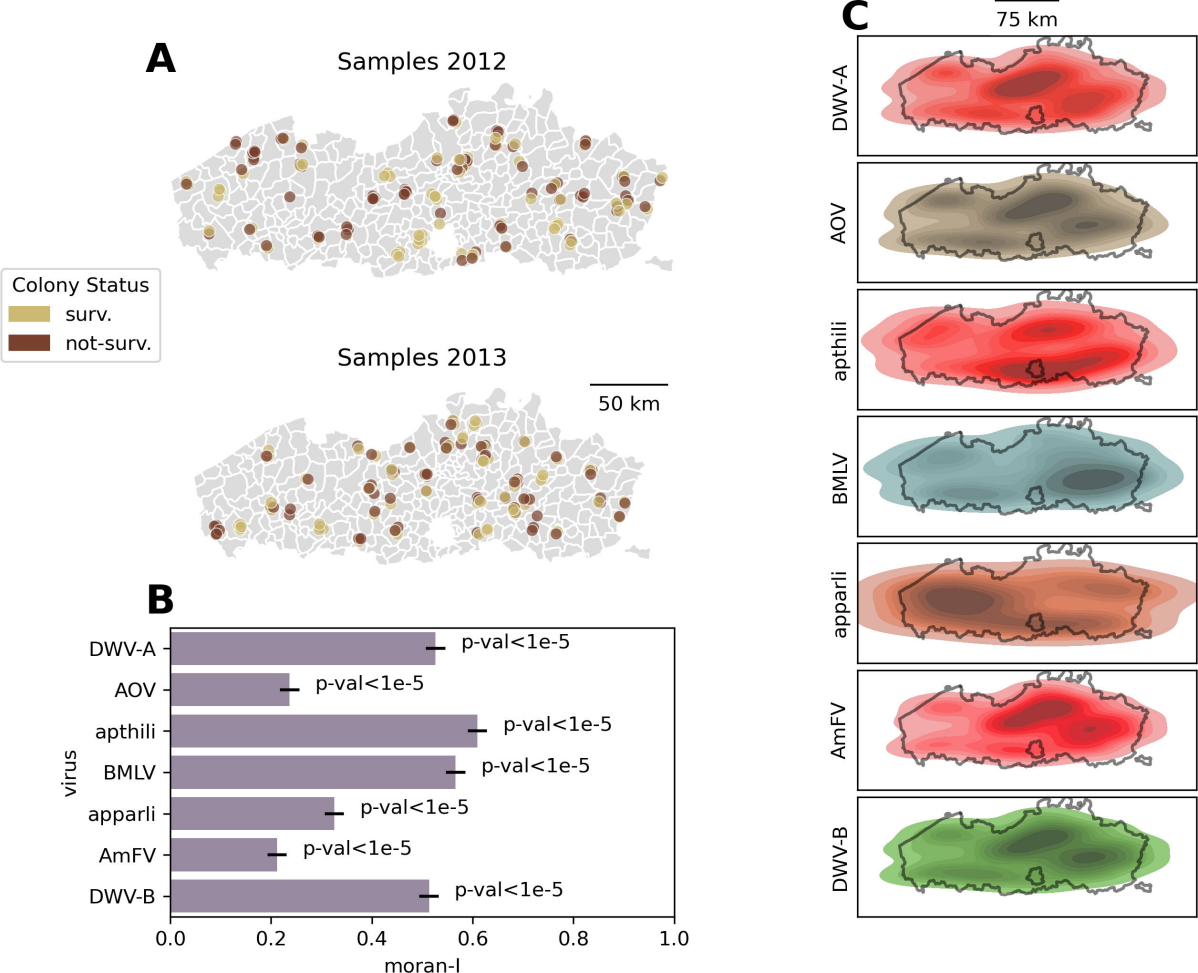
Geospatial analysis samples from Flanders reveal bee viruses follow a clustered pattern of occurrence. (**A**) Different sampled communes are shown for 2012 (top) and 2013 (bottom). Samples from colonies surviving winter are indicated in yellow, those from colonies not surviving winter are indicated in red. Coordinates are jittered along both the lateral and longitudinal direction to better represent different samples taken from the same commune. (**B**) Moran’s I statistic for autocorrelation is shown for the different viruses. Significance values are indicated as text labels. Black horizontal bars show standard deviations. (**C**) Kernel density estimations using viral quantifications as weights are shown for the different viruses. Darker colors indicate higher viral loads.

To investigate any geographical patterns underlying the data, the high-resolution (commune level) spatial metadata collected in the EPILOBEE framework were exploited and integrated with administrative areas from Flanders, Belgium. Samples taken from both years are uniformly distributed over the entire region, and an equal number of samples was taken from winter-surviving and non-surviving colonies (Fig. 3A). Spatial autocorrelation was evaluated by calculating Moran’s I statistic using a weight matrix defined on the commune level and tested for significance using 10,000 permutations. This test indicates if the viral load is spatially randomly distributed (*P*-value >0.05), is spatially dispersed (*P*-value <0.05 and Moran’s I statistic <0), or if spatially clustered (*P*-value <0.05 and Moran’s I statistic >0). All investigated viruses revealed a strong spatially clustered distribution of viral loads (Moran’s I statistic >0, *P*-value <1e-5), with Moran’s I ranging from 0.21 (AmFV) to 0.61 (apthili virus) (Fig. 3B). These results are recapitulated when plotting kernel density estimation over the Flanders region, where all viruses show clear focal points of high viral loads (Fig. 3C).

### Apis orthomyxovirus 1 is present in all bee developmental stages and tagmata as well as in Varroa mites and replicates in adult worker bees

**Fig 4:**
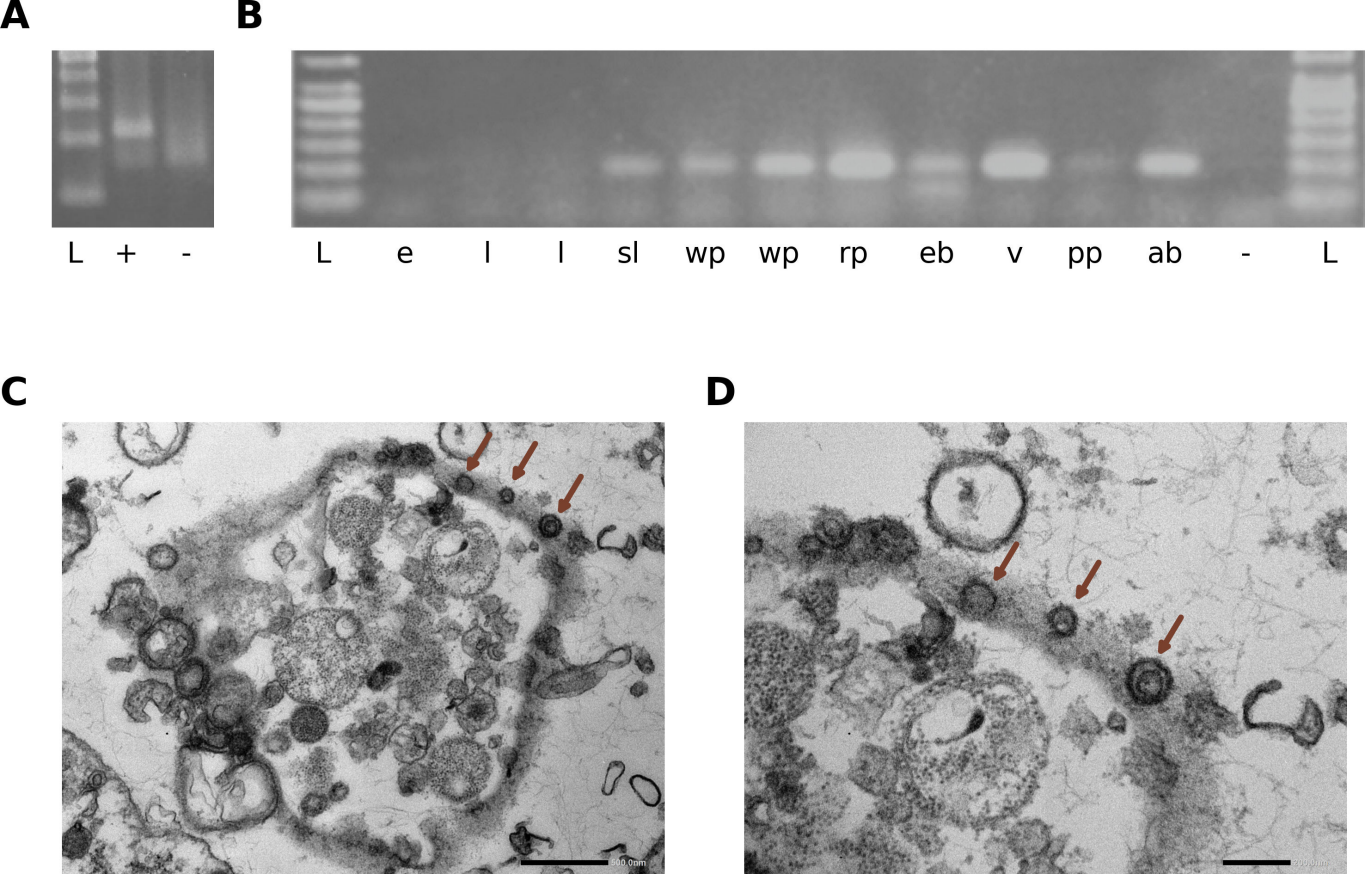
Replication, distribution, and visualization of Apis orthomyxovirus 1. (**A**) Positive-strand detection. Lane L, GeneRuler Marker 100 bp DNA ladder; lane +, PCR-positive worker bee; lane -, negative control. (**B**) RT-PCR Apis orthomyxovirus virus 1 detection in different developmental stages of the honey bee and *V. destructor*. Lanes L and −, GeneRuler Marker 1 kb DNA ladder and negative control, respectively. Lane e, eggs; lanes l, 3-day-old larva; lane sl, stretched larva; lanes wp, white eyed pupa; lane rp, red eyed pupa; lane eb, emerging bee; lane v, Varroa mite; lane pp, parasitized pupa; lane ab, adult bee. (**C** and **D**) Transmission electron microscopy. Ultrastructure of putative Apis orthomyxovirus 1 virions in hemolymph from *A. mellifera* (indicated with brown arrows). Virions are between 100 and 200 nm in diameter at the surface of hemocytes. (**C**) Overview image taken at 25 k magnification (scale bar indicates 500 nm). (**D**) Image taken at 100 k magnification (scale bar indicates 200 nm).

Since Apis orthomyxovirus 1 was previously associated with both *A. mellifera* and Varroa mites (54), an attempt was made to confirm that this virus truly infects honey bees, and was not derived from the environment or potentially any other organism present in our samples. To prove actual replication of Apis orthomyxovirus 1, MLPA was performed to discriminate between the negative-sense ssRNA genome of the virus and the positive-sense RNA formed during active replication in adult worker bees. This experiment showed clear evidence that Apis orthomyxovirus 1 replicates in bees (Fig. 4A). However, tissue specificity was not tested as for the MLPA reaction the RNA of a complete honey bee was used. RT-PCR results show that Apis orthomyxovirus 1 is present in all developmental stages of worker bees in an infected colony (eggs, a stretched larva, white-eyed pupae, a red-eyed pupa, adult worker). The presence of this virus in eggs implies a potential vertical transmission (Fig. 4B), although eggs and larvae generated only weak bands on the agarose gels. Furthermore, both the Varroa mite and the corresponding parasitized pupa tested positive, confirming the results found by Levin et al. (54). This suggests that Varroa may be involved in vectoring Apis orthomyxovirus 1. Furthermore, RT-PCR showed that Apis orthomyxovirus 1 was present in all three tagmata (head, thorax, and abdomen) as well as the hemolymph of adult worker bees. Due to the distinct morphology of orthomyxoviruses, an attempt was made to visualize the virions. For the visualization, we used transmission electron microscopy on hemolymph of adult worker honey bees which were RT-PCR-positive for Apis orthomyxovirus 1. Structures were revealed reminiscent to that of orthomyxoviruses, with a diameter between 100 and 130 nm (Fig. 4C and D). These virions also show spike-like structures on their surface, similar to structures identified from the aransas Bay virus with virions of 95–200 nm in diameter, and with those of the upolu virus, described previously (55). Both these viruses are currently unclassified, but probably belong to the genus *Thogotovirus*.

## DISCUSSION

This study provides insight on the prevalence, yearly difference (2012 versus 2013), and spatial distribution of four well-established (DWV-A, DWV-B, AmFV, and BMLV) and three recently described (Apis orthomyxovirus 1, apthili virus, apparli virus) honey bee viruses in Belgium. These viruses were selected based on the relative abundances from a previous metaviromic survey (30), their biological relevance or their estimated prevalence. Surprisingly, more than 70% of all the tested samples were positive for DWV-A, DWV-B, and AmFV for both tested years. It has to be noted that our approach cannot distinguish between possible DWV-A and DWV-B recombinants. A strong yearly difference (2012 vs 2013) was observed for the newly described apthili virus (Fig. 2A and B). Additionally, the data hint at yearly differences for Apis orthomyxovirus 1, BMLV, and DWV-A, implying that these might undergo yearly differences as well (Fig. 2B). Strikingly, an overwhelming majority of all samples was shown to carry at least one of the investigated viruses, as only in 2013, a few samples were present that did not contain any detectable virus (Fig. 2C). Multiple regression models achieved a moderate accuracy, with a substantially higher sensitivity than specificity (Fig. 2D). This lack of power to detect true negatives could potentially be explained by the influence of other variables that were not measured in this framework (such as temperature, pollution, or pesticides) and the fact that only two bees were sampled per colony which might not represent the viral status of the entire colony (56–60). More notably, despite the relatively large sample size of 300 colonies, the number of individuals per sample (two) is a limitation of this study. Since the individuals taken for this study were different from those investigated in the metaviromic study, we compared the correlations between the normalized metaviromic counts and the absolute values obtained via qRT-PCR in this study. We saw a strong correlation for almost all of the seven specific viruses investigated in both assays, implying that two individuals to some degree reflect the infection status of the worker bees (Fig. S9). How well this represents the viral status of the entire colony is not only dependent on the scrutinized virus but also on where, when, and how a sample is taken. Since little is known about some of the viruses investigated in this report, larger studies with increased sampling frequency, scope, and broader metadata collection could ameliorate these limitations. The high sensitivity (75.5% in the full model) does reflect that a specific viral status (based on the seven viruses included in this study) can at least predict colony winter survival in a subset of colonies. The fact that the observed difference in colony winter survival between 2012 and 2013 in our study (32.4% versus 14.8%, respectively) could not be properly explained by the viral data gathered in this study reinforces this notion. Spatial correlation analyses (Fig. 3) revealed that all investigated viruses were significantly spatially clustered, implying that the vast majority of viral transmission occurs local (on commune level). Additional sampling could elucidate how this local transmission potentially differs between the viruses, for example, between DNA and RNA viruses, or between *V. destructor* vectored viruses and non-vectored viruses. Further evidence is provided that Apis orthomyxovirus 1 is truly infecting honey bees by detecting positive-sense RNA in adult worker bees (Fig. 4B) and by visualizing orthomyxo-like virus-like particles in hemolymph (Fig. 4C). This finding, combined with an estimated prevalence ranging from 10% to 20% implies that Apis orthomyxovirus 1 could be an emerging (or previously overlooked) pathogen in honey bees and should be carefully monitored in the future. This is further stressed by the evidence that not only all developmental stages can be infected, but also *V. destructor* samples were positive [shown here in Fig. 4A and by Levin et al. (54)]. Aside from the observed yearly differences in apthili viral loads, a surprising association with colonies surviving winter was shown, i.e., the viral load of apthili virus was higher in surviving colonies than in not-surviving colonies (Fig. 2A). Whether this virus has a positive effect on bee health or if this association is a consequence of another biological role (such as niche competition or infection exclusion (61) remains unclear. The cause for the striking yearly difference in viral loads of apthili virus and, to some extent, Apis orthomyxovirus 1 and BMLV (Fig. 2A) remains to be elucidated. Additional longitudinal samples (within and between seasons) and more metadata (for example, colony size, temperature, colony activity, how beekeepers replace lost colonies) could provide additional information. Finally, the predicted apparli virus did not reveal any associations with winter survival, nor did it display strong yearly difference and may therefore potentially be asymptomatic. Despite the fact that partiti-like viruses are known to infect plants and fungi, evidence is accumulating that at least some of these viruses can replicate in insects as well (53, 62). The high load of apparli virus in some of the samples, combined with its phylogenetic clustering together with a similar RNA sequence found in honey bees sampled in the USA (AEA40853.1), implies that this is a true insect-specific partiti-like virus rather than a contamination from plants, although further confirmation is needed.

## Data Availability

All source data for this study, as well as the code to generate the statistical analysis and figures, are available on github (https://github.com/matthijnssenslab/Beestat). The original sequencing data for the metaviromic study (30) are available in SRA under accession PRJNA579886. The sequence for BMLV is available in GenBank under accession ID PP057991, the sequence for apthili virus is available in GenBank under accession ID PP057990, the sequence for apparli virus is available in GenBank under accession ID PP057998, and the sequences for Apis orthomyxovirus 1 are available in GenBank under accession IDs PP057992 to PP057997.

## SUPPLEMENTAL MATERIAL

Supplemental figures spectrum.03581-23-s0001.pdf Fig. S1-S9.

Supplemental table spectrum.03581-23-s0002.docx Table S1.

ASM does not own the copyrights to Supplemental Material that may be linked to, or accessed through, an article. The authors have granted ASM a non-exclusive, world-wide license to publish the Supplemental Material files. Please contact the corresponding author directly for reuse.
