# Supplementary material for: Known and novel viruses in Belgian honey bees: yearly differences, spatial clustering, and associations with overwintering loss
Source: Microbiol Spectr. 2024 Jun 11;12(7):e03581-23. doi: 10.1128/spectrum.03581-23 (PMC11218457; doi:10.1128/spectrum.03581-23)
Supplement: Supplemental figures — Fig. S1-S9. [file spectrum.03581-23-s0001.pdf]

Supplemental Figure legends

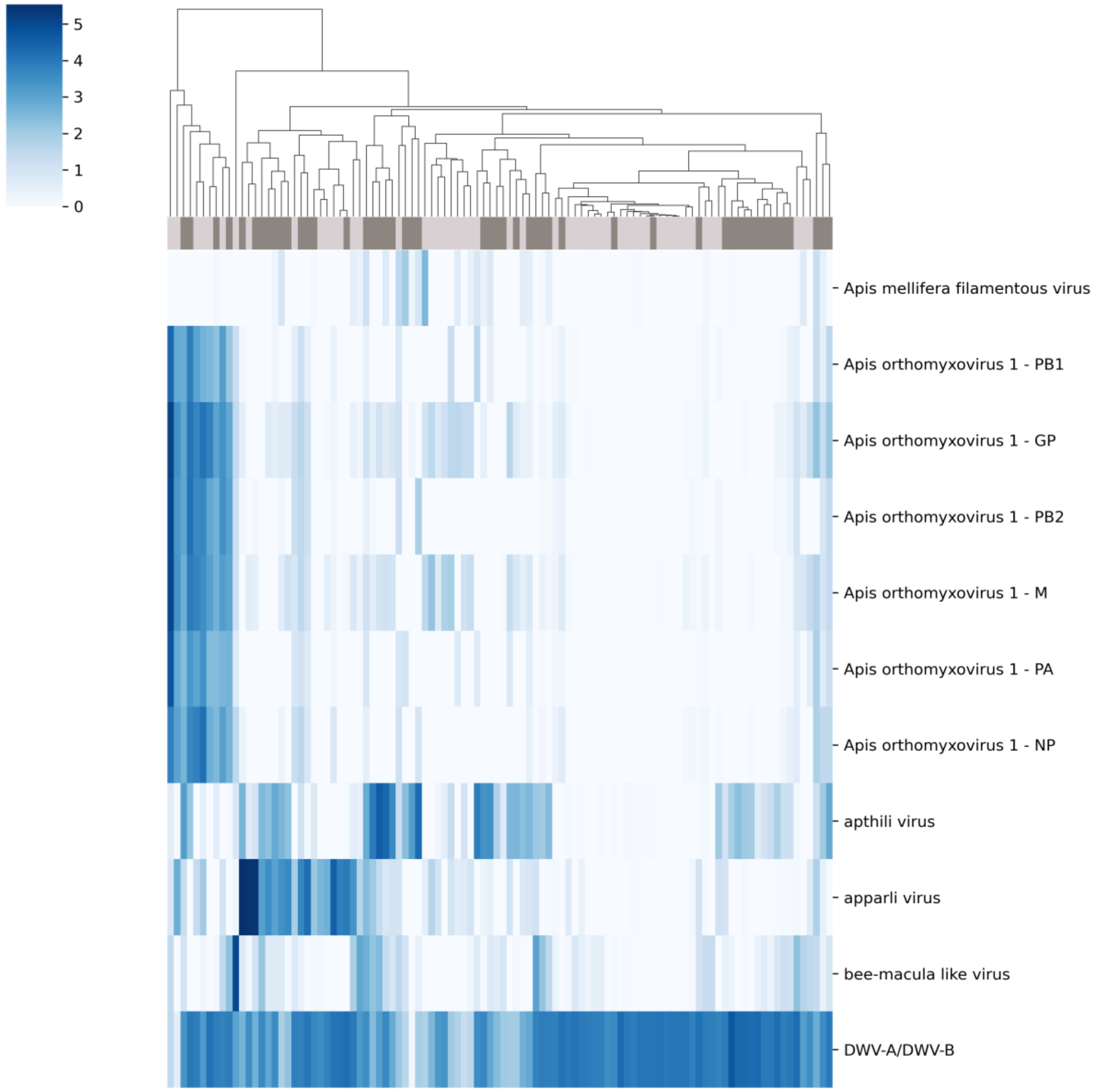

Supplemental Figure S1. Heatmap showing the log-transformed relative abundances for the selected viruses. Each row indicates a selected virus (or a segment of a selected virus in case of Apis orthomyxovirus 1), columns reflect different pools. Sampling years are indicated with an additional row of colours on the top (light grey indicates pools from sampling year 2012, dark grey indicates pools from sampling year 2013).

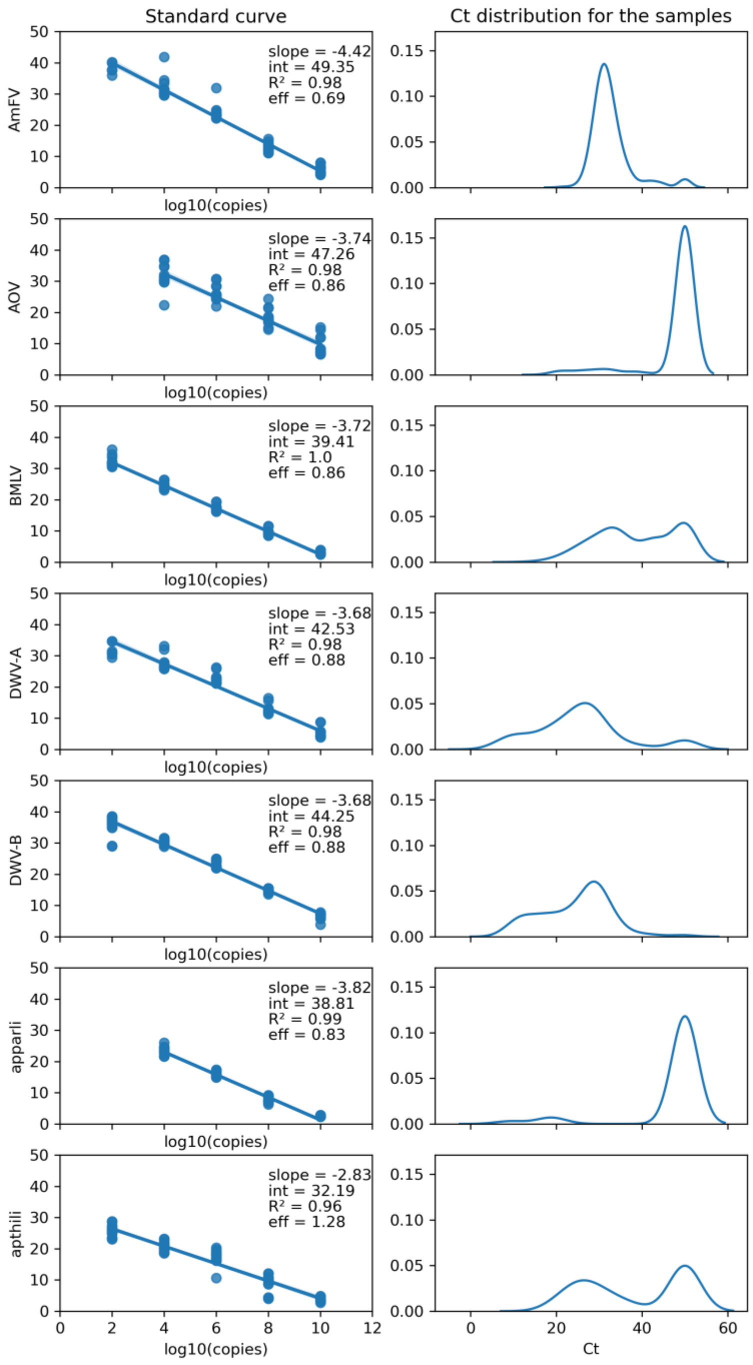

Supplemental Figure S2. Overview of the qPCR performance. Each row indicates a selected virus, the first column shows the standard curve obtained for template copies at different concentrations. For each standard curve, the inferred slope (slope), intercept (int), R-squared value (R^2) and calculated PCR efficiency (eff) are indicated on the plot (left column). The second column shows the density of Ct values obtained for all of the different samples. The Ct value for samples where a Ct value could not be obtained are set to 50 for visualisation purposes.

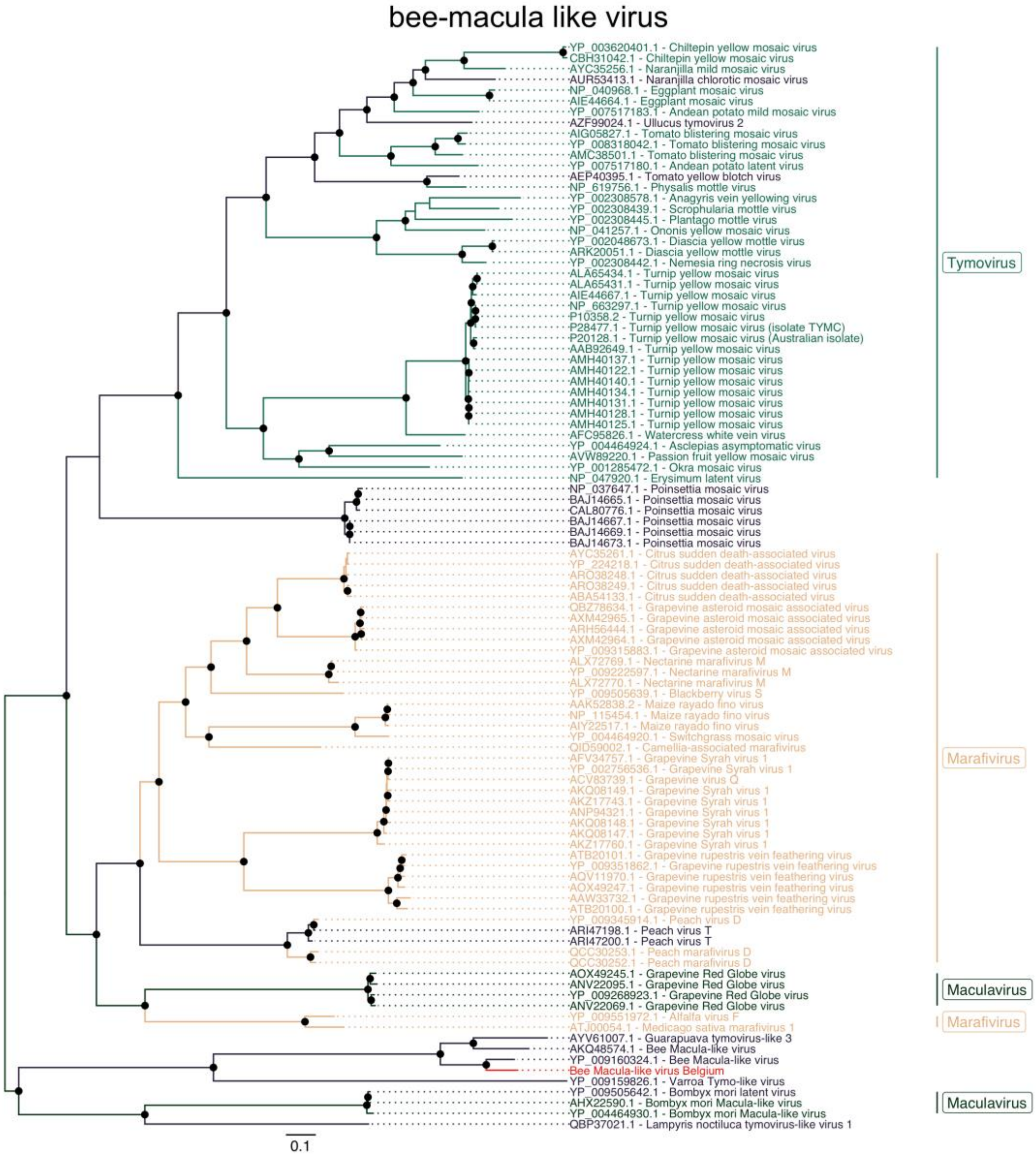

Supplemental figure S3. Maximum-likelihood phylogenetic tree for the retrieved bee macula-like virus. The obtained selected virus is indicated in red. Sequences retrieved from members of the *Tymovirus, Marafivirus* and *Maculavirus* genera are indicated in light green, orange and green, respectively, and annotated with a clade label. Currently unclassified sequences are indicated in grey. Black node labels reflect bootstrap values greater than 70. The tree is based on amino acid alignments of the putative polyprotein.

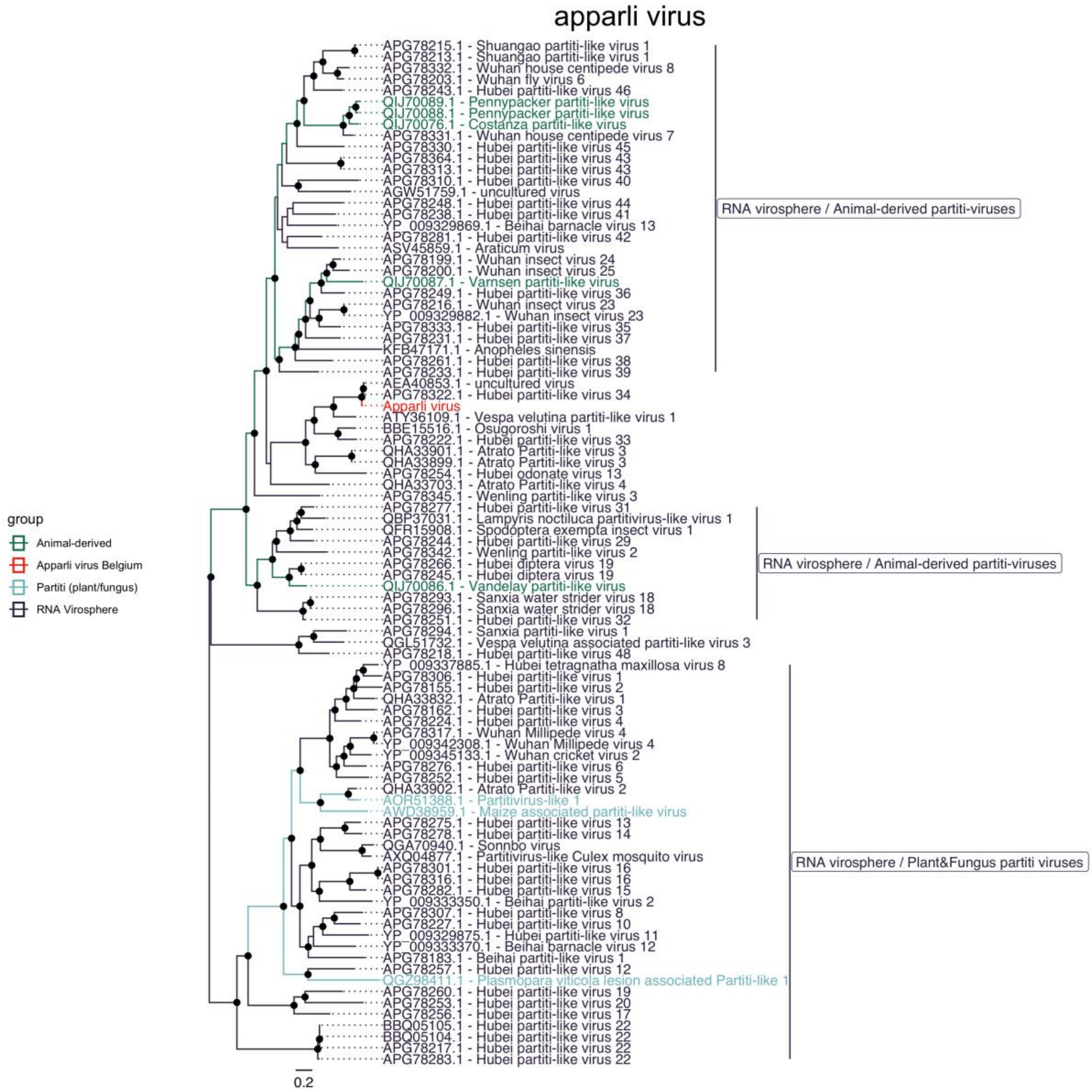

Supplemental figure S4. Maximum-likelihood phylogenetic tree for the retrieved apparli virus. The obtained selected virus is indicated in red. Sequences retrieved from the RNA virosphere (putative insect infecting viruses) study are indicated in dark blue, partiti viruses infecting plants or fungi are indicated in light blue, and animal-associated partiti viruses are indicated in green. Black node labels reflect bootstrap values greater than 70. The tree is based on amino acid alignments of the putative *rna-dependent rna polymerase*.

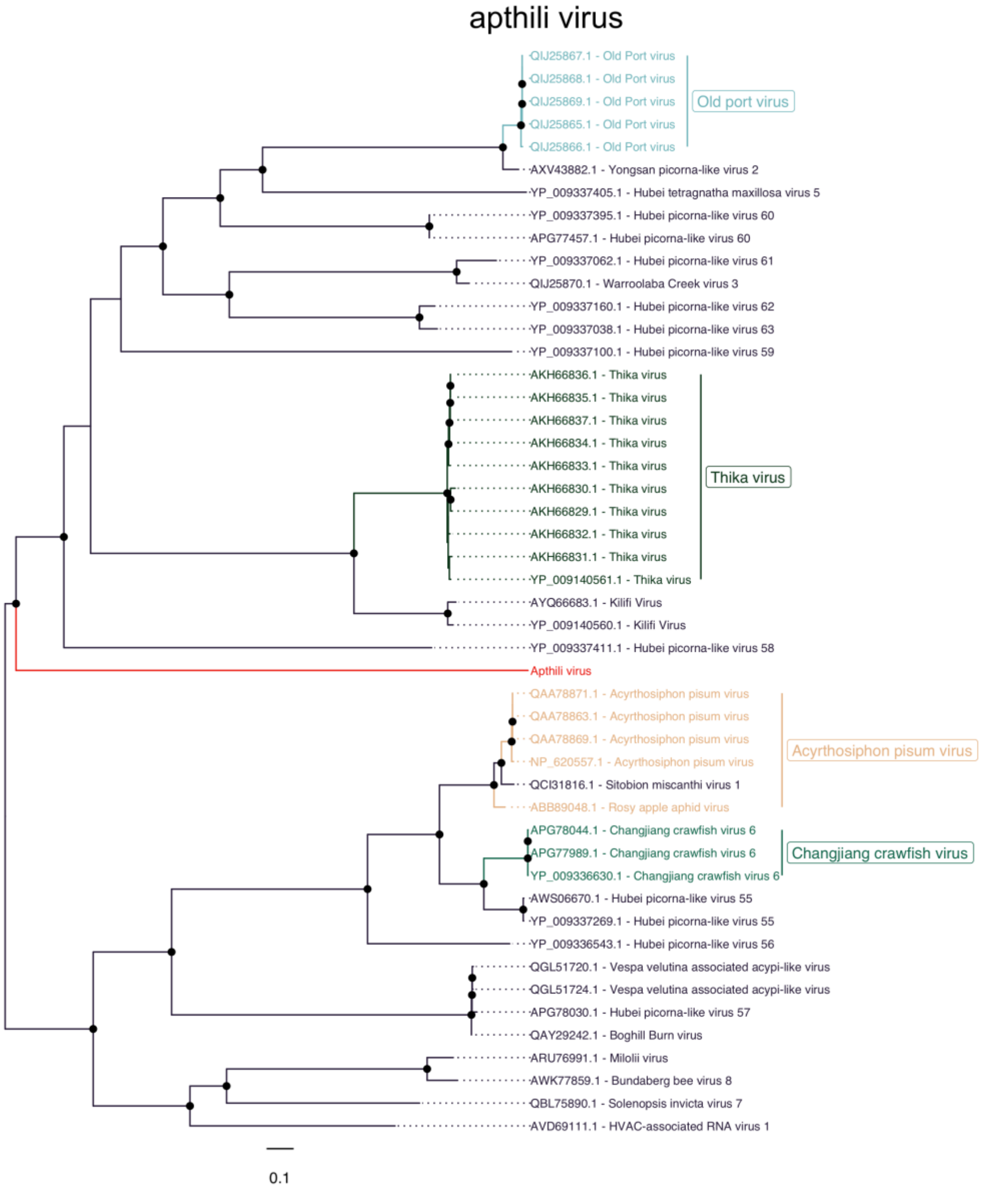

Supplemental figure S5. Maximum-likelihood phylogenetic tree for the retrieved apthili virus. The obtained selected virus is indicated in red. Sequences retrieved from the putative old port virus lineage, thika virus lineage, acyrthosiphon pisum lineage and the changjang crawfish virus lineage are indicated in light blue, dark green, orange and light green, respectively. Black node labels reflect bootstrap values greater than 70. The tree is based on amino acid alignments of the putative *rna-dependent rna polymerase*.

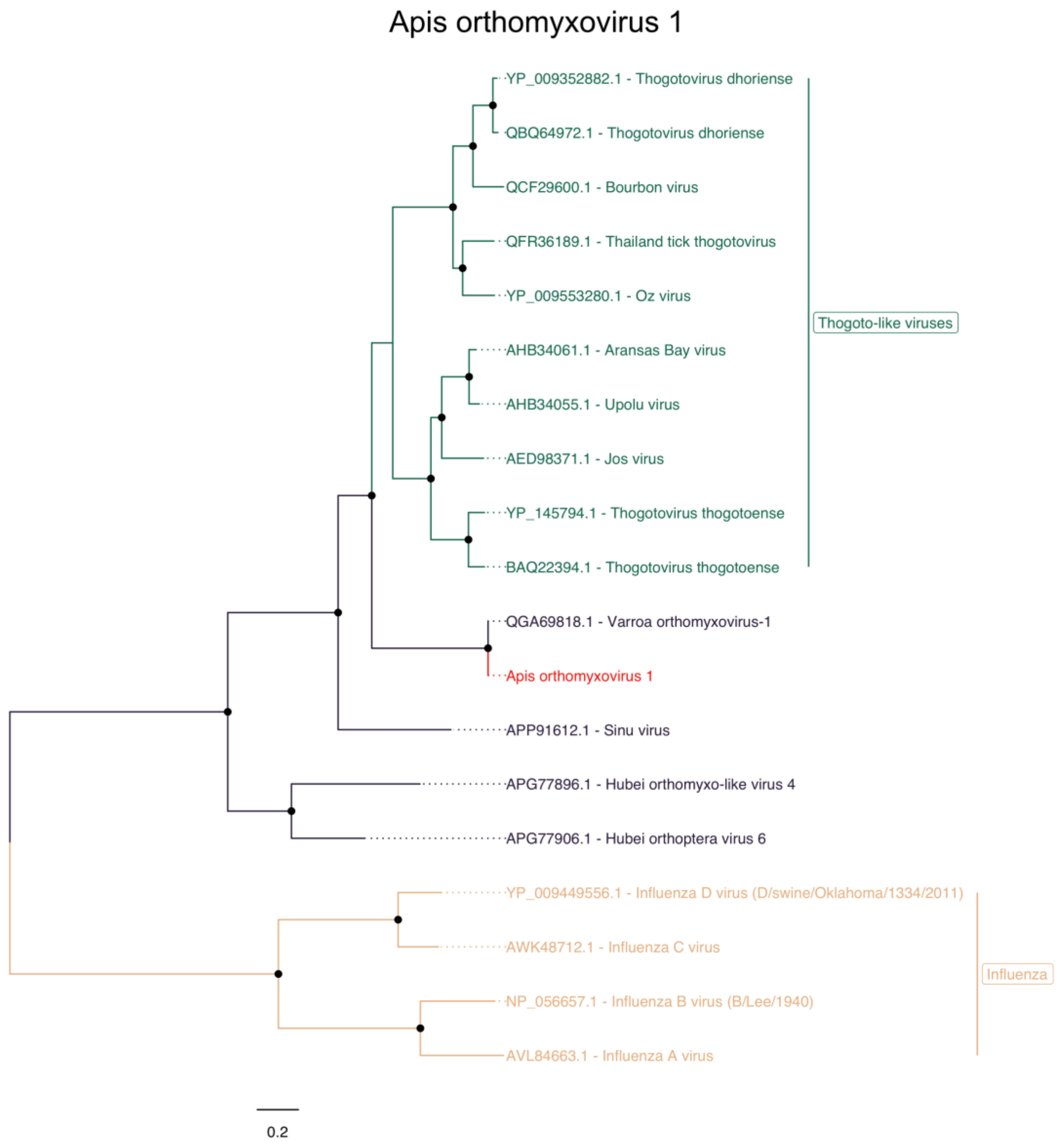

Supplemental figure S6. Maximum-likelihood phylogenetic tree for the retrieved Apis orthomyxovirus 1. The obtained selected virus is indicated in red. Sequences derived from thogoto-like viruses are indicated in green, Influenza viruses are indicated in orange. Unclassified sequences are indicated in grey. Black node labels reflect bootstrap values greater than 70. The tree is based on amino acid alignments of the putative *PB2* segment.

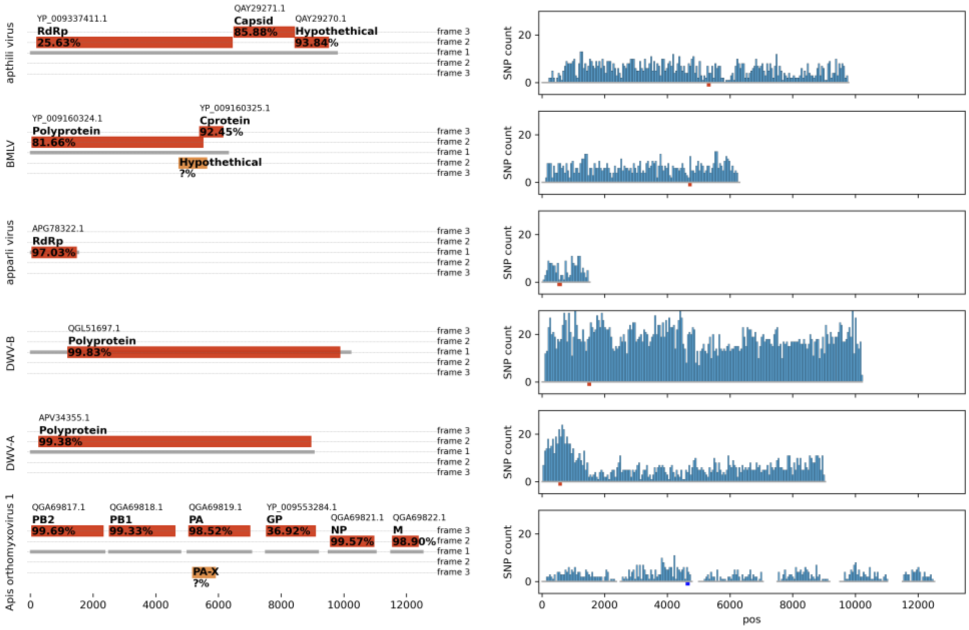

Supplemental figure S7. Putative genomic structure for the selected viruses in this study. Left: Y-axis labels reflect the specific viruses. Sequence length (in base pairs) is indicated on the X-axis. Dark grey rectangles reflect the retrieved nucleotide sequence, associated red (+ frame) or orange (- frame) rectangles indicate the predicted open reading frames. These open reading frames are annotated with their putative predicted function, and percentage of amino acid similarity with their best blast hit. The accession of the best hit is indicated above the function. Predicted open reading frames without any blast hits are indicated with a question mark. Right: Histograms depicting the number of single nucleotide polymorphisms called in the metaviromic data with reference to the consensus sequences. The amplicon used in the qPCR assays is indicated with red rectangles, and were selected in regions with low numbers of SNPs (except for DWV-A and DWV-B, in order to allow their discrimination).

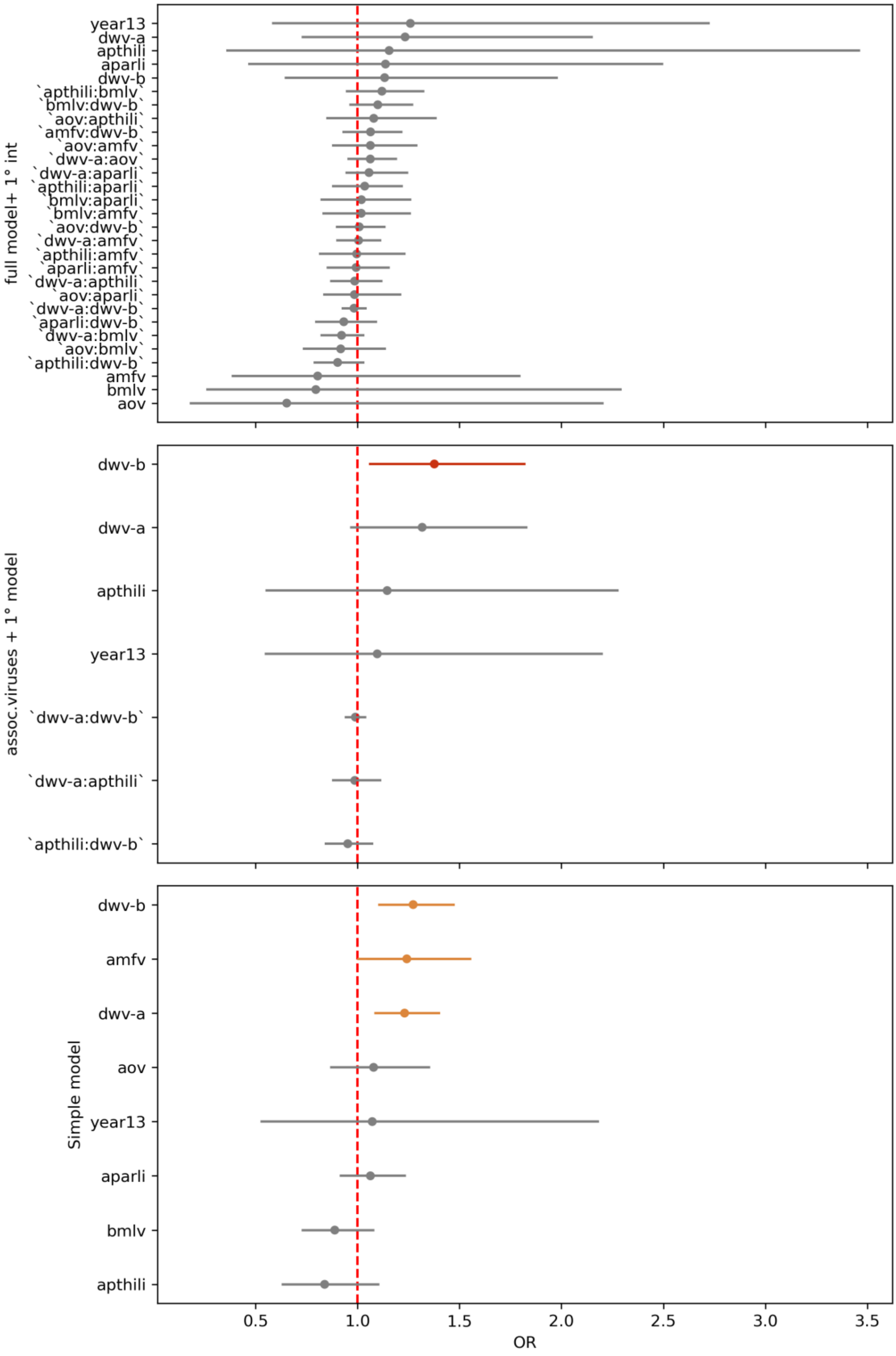

Supplemental figure S8. Multiple logistic regression results for the different models. The x-axis shows the odds ratio depicted for the different coefficients included in either the full model with interaction terms (top row), the reduced model with interaction terms (middle row) or the simple model without interaction terms (bottom row). lines indicate 95% confidence intervals around the estimated odds ratio. Non gray estimates represent significant (p-value < 0.05) coefficients in their respective model.

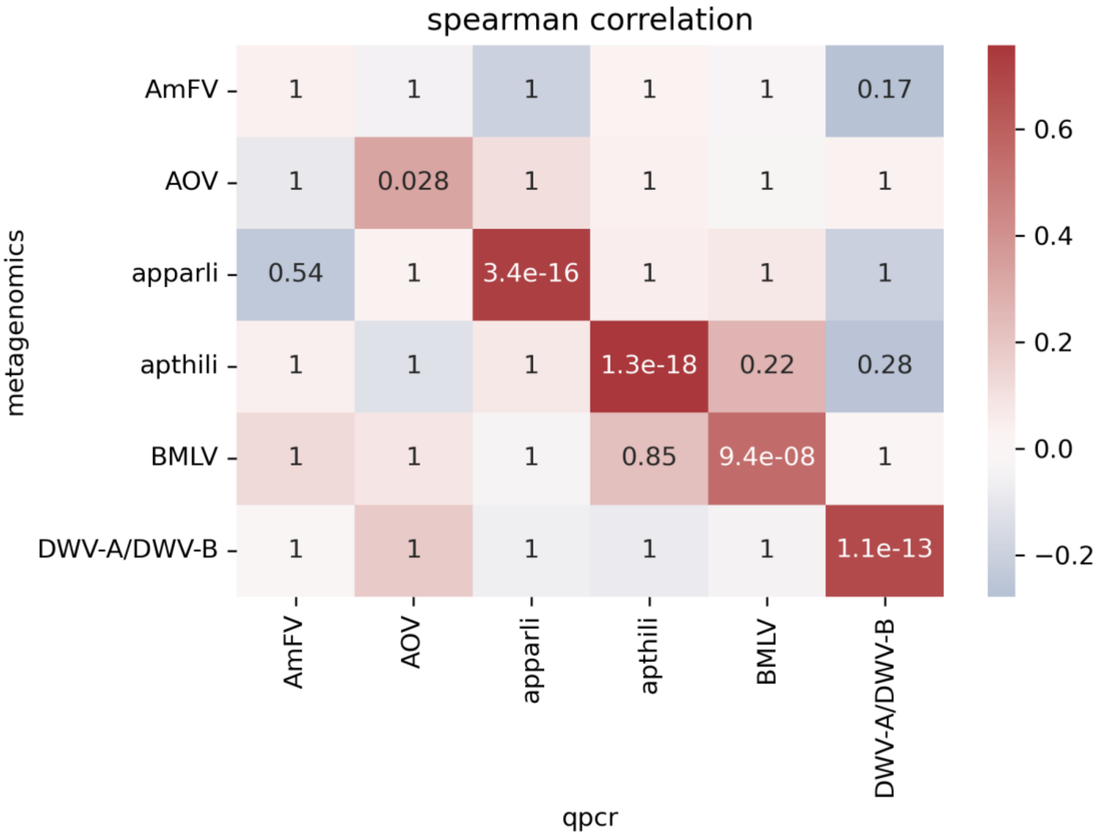

Supplemental figure S9. Spearman correlation between metaviromic relative abundances and absolute virus counts (qPCR). The colours of the heatmap reflect the correlation coefficient between viruses quantified by qPCR (x-axis) and normalised counts from the metaviromic study (y-axis). The values indicated inside the tiles represent bonferroni corrected p-values associated with the correlation coefficient.
