# Supplementary material for: Known and novel viruses in Belgian honey bees: yearly differences, spatial clustering, and associations with overwintering loss
Source: Microbiol Spectr. 2024 Jun 11;12(7):e03581-23. doi: 10.1128/spectrum.03581-23 (PMC11218457; doi:10.1128/spectrum.03581-23)
Supplement: Supplemental table — Table S1. [file spectrum.03581-23-s0002.docx]

**Supplemental table 1: Primer sequences and annealing temperatures as used in the qRT-PCR and MLPA assays**

| **Virus** | **Assay** | **Primer** | **Target region** | **Amplicon length** | **Ann. Temp.** | **Sequences** |
| --- | --- | --- | --- | --- | --- | --- |
| apthili virus | qRT-PCR | apthili_fw | polyprotein gene | 91bp | 60°C | ATACTGGGAGATGACATGCG |
|  |  | aphtili_rv |  |  |  | CAAGACCACACTCAGCGAAG |
|  |  | aphtili_probe |  |  |  | TCCGTGTTCTGCCTTCTCTAGGCA |
|  |  | apthili_std |  |  |  | AAGAAGGAAATACTGGGAGATGACATGCGAATCCGTGTTCTGCCTTCTCTAGGCATTACTTTTGAAAGCATGAGGACCGCCTTCGCTGAGTGTGGTCTTGAATGTGTCC |
| Apis orthomyxovirus 1 | qRT-PCR | ortohomyxo_fw | *PB1* gene | 96bp | 50°C | CCTGGCACAGTTAATGAAGC |
|  |  | orthomyxo_rv |  |  |  | GTTTCTCTCTCAACAACTTCTTGG |
|  |  | orthomyxo_probe |  |  |  | CCCTCATTGAAAGATCCAATTTGCCA |
|  |  | orthomyxo_std |  |  |  | AAGTTTCTCTCTCAACAACTTCTTGGTTTCTTCTGGCAAATTGGATCTTTCAATGAGGGTAAAAATCCGGTTATATAAAGCTTCATTAACTGTGCCAGGTATACCAAC |
|  | MLPA | LPO_MLPA | *PA* gene | 109bp | 60°C | GGGTTCCCTAAGGGTTGGAGGTTGAAACTGTGGTGCTTAAAAGACAAGGCCA |
|  |  | RPO_MLPA |  |  |  | /5Phos/CACCGGCAATGATGAGGTCCAAACCATTATGACTCTAGATTGGATCTTGCTGGCAC |
|  |  | RT-primer_MLPA |  |  |  | CGCCGTCTGCATAGAACTTT |
| apparli virus | qRT-PCR | apparli_fw | polymerase gene | 113bp | 60°C | TGTCCCTAAGATCAGAGCGG |
|  |  | apparli_rv |  |  |  | TAAGCAATAGGCGTCTGTCC |
|  |  | apparli_probe |  |  |  | ATGACCCTCGGTGAAGCGCA |
|  |  | apparli_std |  |  |  | TACAGATGATGTCCCTAAGATCAGAGCGGTTTATGGTTATCCAGTTACGATGACCCTCGGTGAAGCGCAATTTGCACTTCCCTTAATTGAAGAATTTAAGAAAGGACAGACGCCTATTGCTTATGGTTTTGA |
| bee macula-like virus | qRT-PCR | bmlv_fw | polymerase gene | 82bp | 55°C | GCTTTCGATCAGAGCCAACATGG |
|  |  | bmlv_rv |  |  |  | CAAGTTGGCGTGGAATGCCT |
|  |  | bmlv_probe |  |  |  | CGCGCTCGAAGACGACCGCCTCG |
|  |  | bmlv_std |  |  |  | CAAGTTGGCGTGGAATGCCTAATTGCAACATCTTTTCGCGCTCGAAGACGACCGCCTCGCCATGTTGGCTCTGATCGAAAGC |
| Apis mellifera filamentous virus | qRT-PCR | amfv_fw | *Ribonucleotide reductase 2* | 105bp | 60°C | GCGCTCGGGTATGCGATTAACGT |
|  |  | amfv_rv |  |  |  | AGATCGCCCGCTTTGTCGCC |
|  |  | amfv_probe |  |  |  | CGTTCTTCGCAAACACGATCACGAGTCG |
|  |  | amfv_std |  |  |  | AGATCGCCCGCTTTGTCGCCGATCCTGCTCCGACGTTCTTCGCAAACACGATCACGAGTCGAGCAACTCTGAATCGTCTCACGTTAATCGCATACCCGAGCGC |
| DWV-A | qRT-PCR | dwv_fw | L protein | 87bp | 60°C | CGTCGGCCTATCAAAGAG |
|  |  | dwv_rv |  |  |  | TTCTTGACCGACCTCGACC |
|  |  | dwv_probe |  |  |  | AATAGGTTTGCTCCACTGGA |
|  |  | dwv_std |  |  |  | CGTCGGCCTATCAAAGAGCATTCTCCTATATCAGTTTCGAATAGGTTTGCTCCACTGGAATTCCTCAAGGTCGAGGTCGGTCAAGAA |
| DWV-B | qRT-PCR | vdv_fw | polymerase gene | 86bp | 55°C | GCCCTGTTCAAGAACATG |
|  |  | vdv_rv |  |  |  | CGCGTCTTTTCCTACCTCAAT |
|  |  | vdv_probe |  |  |  | AATAGATATTCCCCTTTAGA |
|  |  | vdv_std |  |  |  | GCCCTGTTCAAGAACATGTCCCCATTTCAATCAGTAATAGATATTCCCCTTTAGAATCCCTTAAGATTGAGGTAGGAAAAGACGCG |
